# Probing the range of applicability of structure‐ and energy‐adjusted QM/MM link bonds II: Optimized link bond parameters for density functional tight binding approaches

**DOI:** 10.1002/jcc.26830

**Published:** 2022-03-03

**Authors:** Hans Georg Gallmetzer, Thomas S. Hofer

**Affiliations:** ^1^ Theoretical Chemistry Division, Institute of General, Inorganic and Theoretical Chemistry Center for Chemistry and Biomedicine, University of Innsbruck Innsbruck Austria

**Keywords:** QM/MM, link bonds, ab‐initio methods, density functional tight binding, semi‐empirical, amyloid

## Abstract

Optimized link bond parameters for the C_α_—C_β_ bond of 22 different capped amino acid model systems have been determined at SCC DFTB/mio (self‐consistent charge density functional tight‐binding), SCC DFTB/3ob and GFN*n*‐xTB (*n* = 0, 1, and 2) level in conjunction with the AMBER 99SB, 14SB, and 19B force fields. The resulting parameter sets have been compared to newly calculated reference data obtained via resolution‐of‐identity 2nd order Møller–Plesset perturbation theory. The data collected in this work suggests that the optimized values in this study provide a more suitable setup of the QM/MM link bonds compared to the use of a single global setting applied to every amino acid fragmented by the QM/MM interface. The results also imply that a transfer of the ideal link bond settings between different levels of theory is not advised. In contrast, virtually identical parameters were obtained in calculations employing different variants of the AMBER force field. Considering the increasing success of tight binding based approaches being inter alia a results of their exceptional accuracy/effort ratio the provided collection of link atoms parameters provides a valuable resource for QM/MM studies of biomacromolecular systems as demonstrated in an exemplary QM/MM MD simulation of the *β*‐amyloid/Zn^2+^ complex.

## Graphical abstract

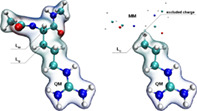

## 1 INTRODUCTION

Nearly five decades after their inception by the influential works of the Nobel laureates Martin Karplus, Michael Levitt and Arieh Warshel^1^, ^2^, ^3^, ^4^, ^5^ mixed quantum mechanical/molecular mechanical (QM/MM) approaches^1^, ^3^, ^6^, ^7^, ^8^, ^9^ still comprise a promising and highly active area of research. While the broad applicability of QM/MM methods has diversified this field into virtually all areas of chemical sciences, a large number of studies is still aimed at the initial applications focused on biomacromolecular systems.^10^ Hybrid QM/MM methods exploit the accuracy of quantum mechanical (QM) methods^11^, ^12^ for the description of the chemical most relevant part while on the other hand less‐accurate but at the same time less‐demanding molecular mechanical (MM) approaches^13^, ^14^ are considered sufficient to represent the remaining part of the system (e.g., the bulk of a liquid or the structure of an entire biomolecule). Thus, in addition to methodical developments associated to the QM/MM hybrid approach such as advanced embedding techniques^7^, ^15^, ^16^ and improved adaptive frameworks,^17^, ^18^ progress achieved in both QM and MM techniques for the description of chemical systems directly contributes to the increasing success of this versatile method.

In particular, research associated with the demanding QM approaches focused on improving the accuracy while keeping the computational demand manageable resulted in a hierarchy of increasingly complex methods. While high‐level QM approaches such as density functional theory (DFT)^19^, ^20^ and even post‐Hartree Fock methods^11^, ^12^ can be routinely applied in QM/MM simulations, the associated computational effort imposes limitations in both the treatable system size as well as the achievable number of simulations steps, for example, when applying the QM/MM framework in the context of Monte‐Carlo and molecular dynamics (MD) simulations.^21^, ^22^, ^23^ A possible alternative enjoying widespread application in QM/MM studies are semi‐empirical QM methods.^24^, ^25^ By introducing various approximations based on DFT or/and Hartree‐Fock (HF) theory a more efficient yet oftentimes less accurate description of molecular interactions is achieved. One increasingly successful family of semi‐empirical approaches is density functional tight binding (DFTB).^26^, ^27^, ^28^, ^29^, ^30^ These methods exploit the efficiency of tight binding (TB) theory^31^ but maintain their accuracy via a parametrization against more demanding DFT approaches. Despite their semi‐empirical character DFTB methods have been applied with large success in QM/MM simulations of various chemical systems.^32^, ^33^, ^34^, ^35^

One particular challenge in a QM/MM simulation is linked to the partitioning of the system into a QM and MM region which in the case of macromolecular systems involves the fragmentation of chemical bonds separated by the QM/MM interface. A variety of approaches for the treatment of QM/MM frontier bonds such as pseudobond^36^, ^37^ and quantum capping potential approaches,^38^, ^39^ adjusted connection atoms,^40^ effective core potential techniques,^41^, ^42^ effective group potentials^43^ and the structure‐dependent effective Hamiltonian^44^ method have been developed, with the link‐atom technique^45^, ^46^ being one of the most widely employed approaches. This is inter alia due to the fact that no modification of the employed quantum mechanical routines is required, making this method particularly flexible when changing the theoretical level of theory which may be associated with a change of the applied QM calculation package, for example, when comparing results obtain via perturbation theory to those calculated via DFTB as done in this work.

**FIGURE 1:**
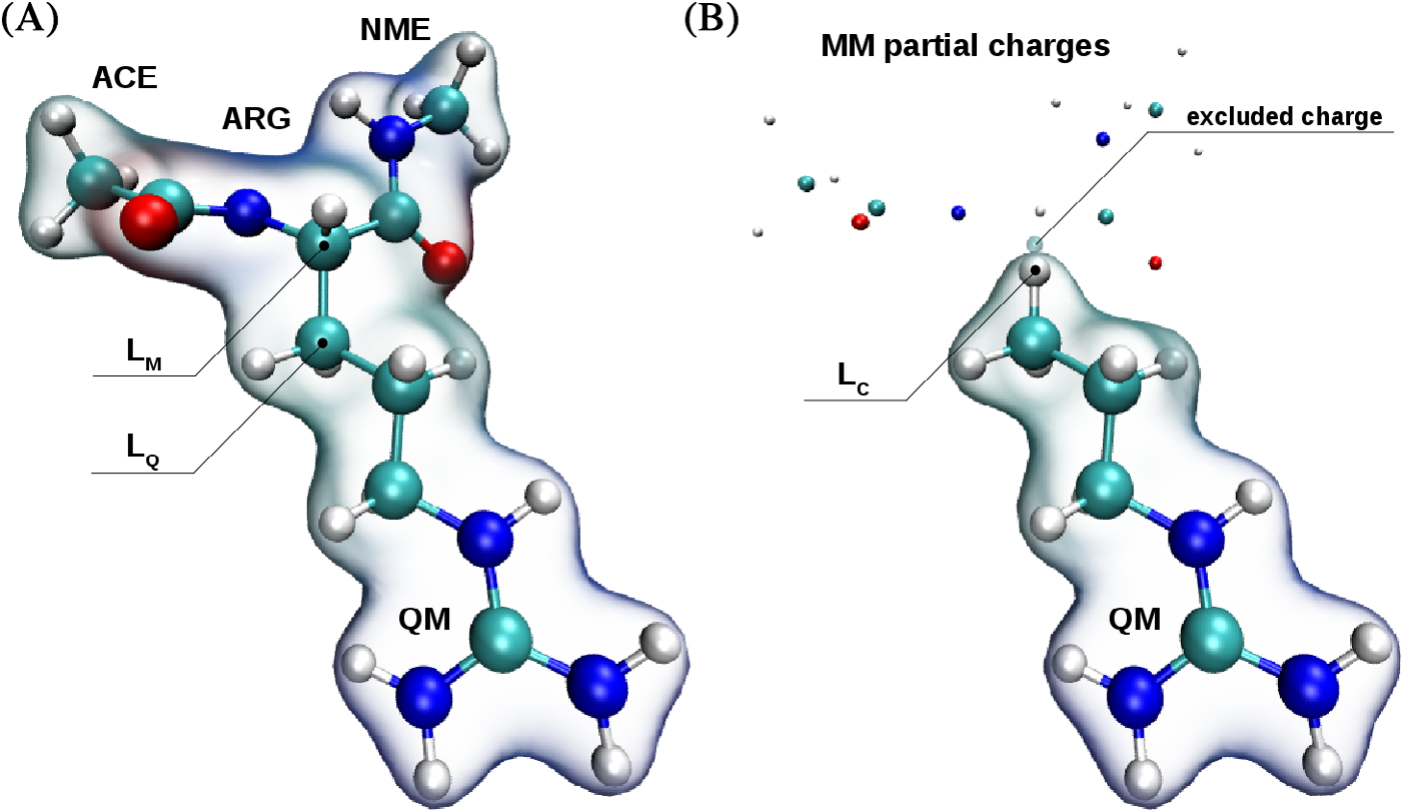
Link bond definition in the ACE‐ARG‐NME system for (A) the full QM reference and (B) the QM/MM setup. In the latter case, only the side chain of ARG was included in the QM‐treatment employing hydrogen as capping atom L_C_. Typically, the partial charge of L_M_ is excluded when applying electrostatic embedding in the QM calculation due to its vicinity to L_C_. However, L_M_ is fully considered when evaluating all other contributions in the system including also the non‐coulombic QM/MM coupling potential

In the link‐atom framework, the valence introduced by fragmenting the QM/MM frontier bond L is compensated via the introduction of a suitable capping atom L_C_ (see Figure 1). In the majority of cases, a hydrogen atom is employed, which is a suitable choice if the bond in question displays an apolar character. Although this capping atom introduces additional degrees of freedom, its location depends strictly on the positions of the QM and MM parent atoms L_Q_ and L_M_ (see Figure 1). While some approaches consider a fixed distance between L_Q_ and L_C_ irrespective of the actual L_Q_—L_M_ bond length, a more adequate approach^47^, ^48^ is the use of a distance ratio *ρ*_*Link*_ according to

(1) $$r_{L_{C}}=\rho_{\text{Link}}\cdot \left(r_{L_{M}}-r_{L_{Q}}\right)+r_{L_{Q}}$$

with **r** being the position of the respective atom. This ensures that any variation in the parent L_Q_—L_M_ bond is replicated proportionally by the L_Q_—L_C_ bond. In addition, this linear relationship enables a re‐distribution of any force contribution **F** acting on L_C_ to the atoms of the host bond via

(2) $$F_{L_{M}}=-\frac{\partial \left\langle \Psi |\hat{H}|\Psi \right\rangle }{\partial r_{L_{C}}}\cdot \frac{\partial r_{L_{C}}}{\partial r_{L_{M}}}=F_{L_{C}}\cdot \rho_{\text{Link}}$$

(3) $$F_{L_{Q}}=-\frac{\partial \left\langle \Psi |\hat{H}|\Psi \right\rangle }{\partial r_{L_{C}}}\cdot \frac{\partial r_{L_{C}}}{\partial r_{L_{Q}}}=F_{L_{C}}\cdot \left(1-\rho_{\text{Link}}\right)$$

thereby eliminating any additional degrees of freedom arising due to the introduction of the capping atom L_C_.

**FIGURE 2:**
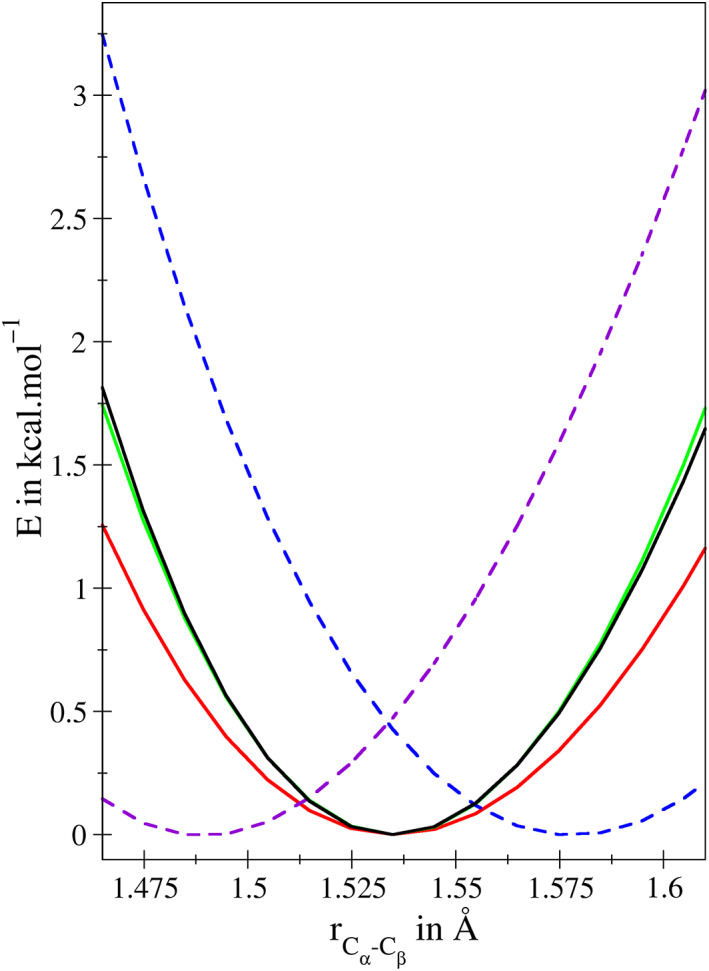
Total energy obtained via a potential energy scan in case of the ACE‐ARG‐NME model system depicted in Figure 1 about the equilibrium distance of the C_α_—C_β_ bond at RIMP2/cc‐pVTZ level. In order to achieve a correct representation of the full QM reference (black) in a QM/MM calculation employing the AMBER 14SB force field an ideal placement of the capping atom using a *ρ*_*Link*_‐value of 0.723 (red) is required. To compensate the difference in bond strength between the C—H link bond and the C_α_—C_β_ parent bond, a harmonic potential with reduced force constant of 200 kcal mol^−1^ Å^−2^ has to be applied (green) resulting in a near‐perfect representation of the QM reference close to the equilibrium. The dashed lines demonstrate the impact of employing non‐ideal *ρ*_*Link*_‐values of 0.700 (purple) and 0.750 (blue), respectively

While this implementation of the link‐bond enables a more seamless integration of the QM/MM boundary into the QM treatment, it is vital to place the capping atom at a chemically reasonable distance from L_Q_.^47^ This is demonstrated in Figure 2, comparing the potential observed for an energy‐minimized ACE‐ARG‐NME model system upon bond stretch along the C_α_—C_β_ bond obtained via an all‐QM and a QM/MM treatment at resolution‐of‐identity Møller–Plesset perturbation theory of second‐order (RIMP2) in conjunction with the AMBER 14SB force field.^49^ In the QM/MM case only the side chain of ARG is included in the QM treatment, however, the influence of the MM partial charges was accounted for utilizing electrostatic embedding.^7^ Due to the close vicinity of L_M_ to the capping atom this partial charge is typically omitted in the embedding, whereas L_M_ is fully considered when evaluating all other interactions in the system including also all non‐coulombic QM/MM coupling contributions. Figure 2 shows that arbitrary selections for the distance ratio *ρ*_*Link*_ (in this example 0.700 and 0.750) result in a shift of the potential along the x‐axis compared to the all‐QM reference. These erroneous QM/MM link bond settings may lead to undesirable behavior in the simulation, e.g. large force components arising from the re‐distribution of $F_{L_{C}}$ although the system should be at its equilibrium. However, when employing an optimized ratio of *ρ*_*Link*_ = 0.723, the equilibrium distance *r*_*eq*_ coincides with that of the all‐QM reference calculation. Considering that a C—H bond displays a weaker bond force constant compared to its C—C counterpart, it is required to add an additional harmonic potential to the parent L_Q_–L_M_ bond. However, the associated force constant *k*_*Link*_ should not correspond to that of a C—C bond but only compensate for the observed difference in the bond strength between the C—C parent bond and its C—H analogue. In this particular example, *k*_*Link*_ amounts to 200.0 kcal.mol^−1^ Å^−2^ while the respective force constant obtained from the all‐QM treatment yields a typical value for a C—C bond of 658.6 kcal.mol^−1^ Å^−2^. The actual value of *k*_*Link*_ thus depends both on (i) the bond strength of the parent bond as well as (ii) the description of the C—H link bond and it can be expected that *k*_*Link*_ may show large deviations when comparing different levels of theory. Finally, when executing the same potential energy scan considering now the three optimized parameters {*r*_*eq*_, *ρ*_*Link*_, *k*_*Link*_}, an ideal representation of the QM/MM link bond with respect to the all‐QM reference is achieved (see Figure 2).

A natural choice for the link‐bond in simulations of peptides and proteins is the apolar C_*α*_—C_β_ bond, thereby including the entire side chain of an amino acid (AA) into the QM region as done in the example above. In this case, a range of 0.709 for the value of *ρ*_*Link*_ has been recommended,^50^ although it was noted by the authors that the ideal setting is strongly dependent on the chosen level of theory. A previous analysis of the link atom properties {*r*_*eq*_, *ρ*_*Link*_, *k*_*Link*_} for ACE‐AA‐NME model systems employing different DFT flavors has demonstrated that the ideal distance ratio *ρ*_*Link*_ determining the placement of the link atom is highly sensitive to both the nature of the amino acid as well as the applied level of theory.^47^ Considering the increasing success of DFTB‐based methods in QM/MM simulations of biomolecular systems, a comparative analysis yielding adequate link‐bond parameters {*r*_*eq*_, *ρ*_*Link*_, *k*_*Link*_} for each amino acid appears to be highly beneficial to enhance the accuracy of QM/MM simulations of proteins and peptides. Thereby, also different protonation states should be considered, as for instance relevant in the histidine residues HID and HIE representing protonation at the *δ*‐ or *ϵ*‐N atom, respectively. In addition, the extraordinarily beneficial cost/accuracy ratio of DFTB approaches also enables the assessment to what extend the link atom parameters depend on the nature of the applied force field, which has not been investigated in previous studies.

In this work ideal link atom parameters for 22 different amino acid residues following the AMBER force field definitions^49^, ^51^, ^52^ have been determined at self‐consistent charge density functional tight‐binding (SCC DFTB) level utilizing the 3ob^53^ and mio^28^, ^54^ parameter sets as well as via the extended tight binding for geometries, frequencies and non‐bonded interactions (GFN*n*‐xTB, *n* = 0, 1, 2) framework.^55^, ^56^, ^57^ The results have been compared against high‐level QM reference data obtained at the correlated ab initio level RIMP2. To assess the impact of the nature of the MM model all calculations have been performed using the AMBER 99SB,^51^ AMBER 14SB,^49^ and AMBER 19SB^52^ parameterizations. Although the considered force fields are all from the same family, their parameterization differs in the assigned atomic partial charges, the Lennard–Jones parameters as well as the treatment of the bonding interactions, in particular the dihedral degrees of freedom. Where applicable the considered amino acid residues also take different protonation states into account.

## 2 METHODOLOGY

Link bond parameters for all amino acids with the exception of proline and glycine have been calculated at different theoretical levels of theory. In the case of histidine, the *δ*‐ and *ϵ*‐isomers HID and HIE differing in the protonation of the N‐atoms in the imidazole moiety have been considered. Similarly, ASH and GLH represent the protonated variants of ASP and GLU, respectively, while LYN corresponds to the deprotonated version of LYS.

**FIGURE 3:**
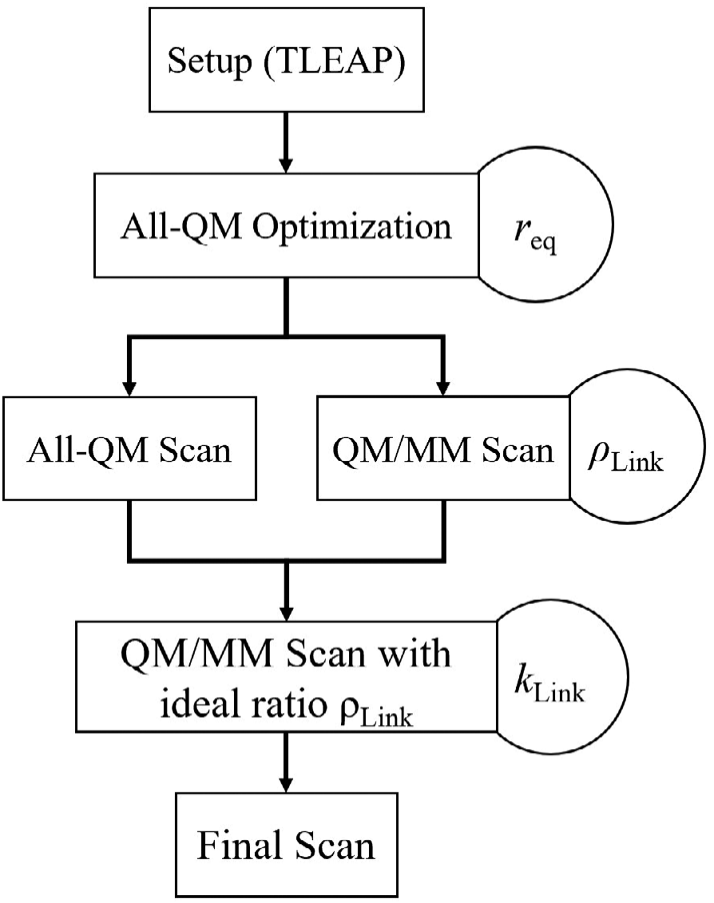
Flowchart depicting the individual steps of the link atom parametrization: Following the generation and subsequent all‐QM optimization of the initial structure yielding the equilibrium bond length *r*_eq_, the all‐QM and QM/MM potential energy scans are carried out. Based on the respective difference measured via the respective RMSD value, the ideal placement of the capping atom *ρ*_Link_ is determined for the particular system. Execution of the potential energy scan using the ideal *ρ*_Link_‐value provides access to the respective link bond force constant *k*_Link_. The final potential energy scan employing the fully parametrized link bond is only required for validation and visualization

An overview of the individual steps of the fully automated parametrization strategy is given in Figure 3. In the first step the initial structure of a particular model system containing the aminoacid capped by N‐terminal acetyl (ACE) and C‐terminal N‐methyl amide (NME) was generated using the program tleap, which is part of the AMBER program package.^58^ This initial configuration was then subjected to an energy minimization using the respective QM method. This step already provides access to the equilibrium distance of the link bond *r*_*eq*_. Following the structure optimization, a potential energy scan along the C_α_—C_β_ bond has been carried out using 10 steps in increments of 0.01 Å in each direction. The resulting potential serves as the reference for the link atom parametrization.

Next, the QM/MM treatment was invoked to determine the ideal placement of the capping atom L_C_ from the C_β_ atom yielding the ideal distance ratio *ρ*_*Link*_. By variation of *ρ*_*Link*_ in the range from 0.69 up to 0.87 in increments of 0.025 and repeated execution of the same potential energy scan at the QM/MM level an RMSD value characterizing the deviation from the all‐QM reference is obtained. The ideal value for *ρ*_*Link*_ is then obtained by locating the respective minimum of the RMSD via cubic spline interpolation.

In the final step, the QM/MM potential energy scan is repeated using the ideal ratio *ρ*_*Link*_. The difference between the resulting potential energy and the all‐QM reference potential enables the determination of the force constant *k*_*Link*_ to compensate the difference in bond strength between the C_β_—H link bond and the respective parent C_α_—C_β_ bond.

All potential energy scans at all‐QM and QM/MM level were performed with the in‐house developed QM/MM program^59^, ^60^, ^61^, ^62^ interfaced to the respective quantum chemical software package. The RIMP2^63^, ^64^ calculations have been carried out using Turbomole 7.5.0^65^ employing the cc‐pVXZ (X = D,T) basis sets^66^, ^67^ in conjunction with the associated auxiliary bases^68^, ^69^ obtained via the EMSL basis set exchange.^70^, ^71^ The DFTB+ package^54^, ^72^, ^73^, ^74^, ^75^ was employed to carry out all SCC DFTB calculations employing the 3ob^53^ and mio^28^, ^54^ parameter sets. All GFN*n*‐xTB (*n* = 0, 1, 2)^76^ calculations have been performed using the xTB software.^55^, ^56^, ^57^

## 3 RESULTS

In the following the individual results obtained for the link bond parameters {*r*_*eq*_, *ρ*_*Link*_, *k*_*Link*_} determined for the 22 considered amino acids model systems at 7 different levels of theory in conjunctions with 3 different AMBER force field parameterizations are compared. The parameters obtained at the different theoretical levels determined in this work are listed in the Supplementary material Tables S1–S7. These collected data are the results of extensive computations based on a total of 462 all‐QM and more than 34,000 QM/MM potential energy scans along the C_α_—C_β_ bond of the individual ACE‐AA‐NME model systems.

### 3.1 Link bond parameters

**FIGURE 4:**
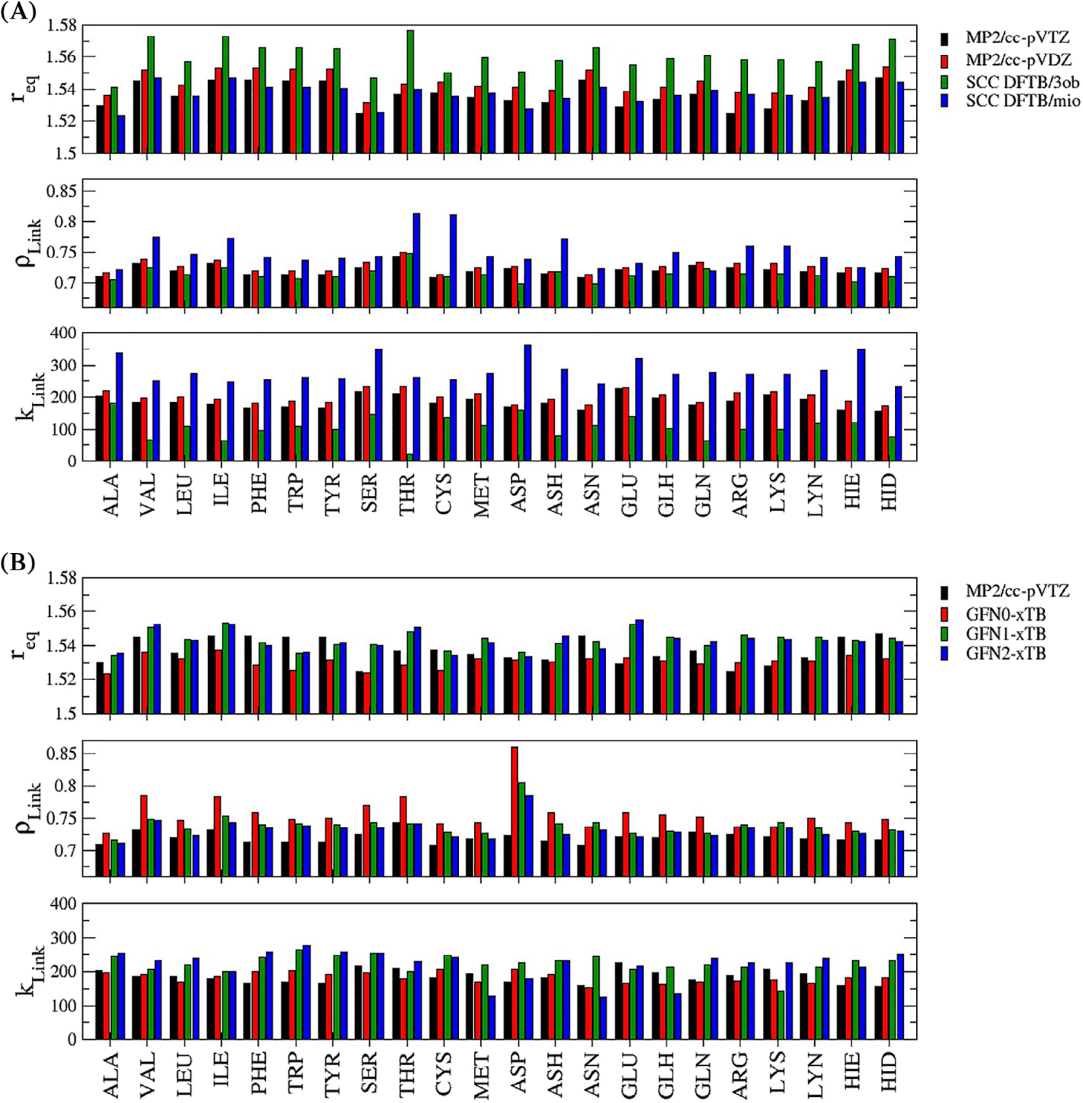
Comparison of the link bond parameters *r*_eq_ in Å, *ρ*_Link_ and *k*_Link_ in kcal mol^−1^ Å^−2^ determined for the 22 considered ACE‐AA‐NME model systems at different levels of theory in conjunction with the AMBER 19SB force field

A comparison of the link bond parameters determined at the 7 different levels of theory in conjunction with the AMBER 19SB parametrization is provided in Figure 4A and B. A summary of the smallest and largest obtained values for {*r*_*eq*_, *ρ*_*Link*_, *k*_*Link*_} along with the respective averages and standard deviations is given in Table 1. Considering that RIMP2/cc‐pVTZ is the most accurate level of theory employed in this study, it serves as the reference in the following discussion. Already when comparing this high‐level data to the RIMP2/cc‐pVDZ results (Figure 4A) small differences in the link bond parameters can be observed, with the average of the three parameters being increased by 0.008 Å, 0.006 and 15.6 kcal.mol^−1^ Å^−2^, respectively. However, the associated standard deviations remained highly similar to the cc‐pVTZ reference.

**TABLE 1:** Smallest and largest obtained values for *r*_*eq*_ in Å, *ρ*_*Link*_ and *k*_*Link*_ in kcal mol^−1^ Å^−2^ along with the respective averages and standard deviations obtained at different levels of theory in conjunction with the AMBER 19SB force field

	RIMP2/cc‐pVTZ	RIMP2/cc‐pVDZ	SCC DFTB/3ob	SCC DFTB/mio	GFN0‐xTB	GFN1‐xTB	GFN2‐xTB
$r_{{\mathrm{eq}}_{\min}}$	1.525	1.531	1.541	1.523	1.523	1.534	1.534
$r_{{\mathrm{eq}}_{\max}}$	1.547	1.554	1.577	1.547	1.537	1.553	1.555
$r_{{\mathrm{eq}}_{\mathrm{av}}}$	1.537	1.545	1.561	1.537	1.530	1.543	1.543
$r_{{\mathrm{eq}}_{\text{stdev}}}$	0.007	0.007	0.009	0.006	0.004	0.005	0.006
$\rho_{{\text{Link}}_{\min}}$	0.708	0.714	0.698	0.720	0.726	0.717	0.712
$\rho_{{\text{Link}}_{\max}}$	0.743	0.750	0.748	0.813	0.861	0.804	0.785
$\rho_{{\text{Link}}_{\mathrm{av}}}$	0.720	0.726	0.714	0.750	0.758	0.739	0.733
$\rho_{{\text{Link}}_{\text{stdev}}}$	0.009	0.009	0.011	0.025	0.028	0.017	0.015
$k_{{\text{Link}}_{\min}}$	157.7	172.3	22.3	233.7	152.5	142.8	124.4
$k_{{\text{Link}}_{\max}}$	227.1	232.2	182.3	362.5	207.8	263.2	275.4
$k_{{\text{Link}}_{\mathrm{av}}}$	185.2	199.8	105.2	281.3	182.4	223.8	220.7
$k_{{\text{Link}}_{\text{stdev}}}$	19.2	18.1	35.5	37.8	15.9	25.5	43.0

The comparison of RIMP2/cc‐pVTZ with the two considered SCC DFTB parameterizations 3ob and mio show different trends (Figure 4A). In the case of *r*_*eq*_ the 3ob set shows notably increased values for all amino acids, with the average increase amounting to 0.024 Å and an increased standard deviation of 0.009 Å. The largest deviation has been observed in the case of THR, which deviates by approx. 2.6% from the RIMP2/cc‐pVTZ value. On the other hand, the equilibrium distances observed in the mio parameter set show a near‐perfect agreement with the high‐level reference data. This picture is reversed when comparing the optimized link atom ratios *ρ*_*Link*_ representing the ideal placement of the capping atom. In this case, the SCC DFTB/mio level shows considerably large deviations towards higher values of *ρ*_*Link*_, with notably increased values observed in case of VAL, ILE, THR, CYS, and ASH. In contrast, the 3ob parametrization shows a very good agreement compared to the RIMP2/cc‐pVTZ reference yielding a comparable average and standard deviation.

However, in the case of the link bond force constant *k*_*Link*_ both the 3ob and mio methods show notable differences. As discussed above these deviations are a consequence of the difference in the bond strength of a C—C bond compared to its C—H counterpart, which then determines the actual value of *k*_*Link*_. While the mio parametrization shows significantly larger *k*_*Link*_ values for all investigated systems, the respective values obtained in the 3ob case are lower throughout the entire set. These dramatic differences in the link bond parameters between the different methods clearly demonstrate the need to identify optimized settings for each level of theory since a given parametrization cannot be unconditionally transferred between different calculation methods.

In Figure 4B the high level RIMP2/cc‐pVTZ results are compared to the data obtained for the GFN*n*‐xTB (*n* = 0,1,2) calculations. The latter represents an increasing hierarchy of the xTB method and consequently, consistent trends can be identified for each of the link bond parameters. The simpler GFN0‐xTB formulation consistently yields shorter equilibrium distances than the higher‐order xTB methods, which is also reflected by the respective average values listed in Table 1. However, comparison to the RIMP2/cc‐pVTZ data reveals an inconsistent pattern, that is, for some systems GFN0‐xTB displays the best agreement while in other cases the higher‐ordered xTB flavors provide the best match. On the other hand, the xTB methods show the smallest standard deviation in the equilibrium distance, indicating that the variation in *r*_*eq*_ is the smallest of all investigated methods and thus even smaller than those observed in both RIMP2 cases.

In the case of *ρ*_*Link*_ all three methods tend towards larger values compared to the high‐level reference, with GFN0‐xTB yielding the largest deviations for virtually all tested systems. It should be noted that in contrast to the RIMP2 and SCC DFTB results, all three xTB variants yield significantly larger *ρ*_*Link*_ values in the case of the ACE‐ASP‐NME system. Visual inspection of the respective minimum configurations did not reveal any particular structural differences to the other levels of theory. However, ASP is one of the smallest amino acids with the respective hydroxy group being in close vicinity to the backbone of the model system. Although the formation of an H‐bond between the OH‐group and H‐bond acceptors in the backbone could be avoided by choosing a suitable initial structure, it appears that the short length of this particular side chain represents a challenging case in the xTB approach. Again the trends observed for the individual systems is also reflected by the associated average values, and the overall high standard deviations can be explained by the large deviation introduced by the ASP system which clearly represents a methodical outlier.

In contrast to the DFTB methods the QM/MM link bond force constants *k*_*Link*_ show a quite good agreement between the individual xTB levels, with GFN1‐ and GFN2‐xTB both showing a trend towards larger values and larger standard deviations. On the other hand, the force constants determined for GFN0‐xTB appear to be in very good agreement with the RIMP2/cc‐pVTZ reference.

The comparison of the results show that the ideal QM/MM link bond parameters {*r*_*eq*_, *ρ*_*Link*_, *k*_*Link*_} (i) depend strongly on the applied quantum chemical calculation method and (ii) show large variations between the individual amino acids. While the ideal values in an actual QM/MM simulation of biomolecule may be further influenced by the instantaneous chemical environment, the use of the optimized parameters derived in this study certainly provide a more suitable setting for the QM/MM frontier bonds over the application of a single global setting applied to all amino acids in agreement with the conclusion given in previous work.^47^, ^48^

### 3.2 Influence of the force field

Since the SCC DFTB and GFN*n*‐xTB methods are much less demanding in their execution compared to other quantum chemical approaches such as density functional theory, it was possible to evaluate the impact of variations in the force field which could not be tested in previous studies.^47^, ^48^ In the case of the considered ACE‐AA‐NME model systems both capping groups and the amino acid backbone are included in the MM zone and influence the atoms of the side chain included in the QM region via (i) the electrostatic embedding treatment, (ii) the associated non‐coulombic QM/MM coupling interactions, and (iii) bonded contributions crossing the QM/MM interface altering the individual minimum configurations. Although the considered parameterizations AMBER 99SB, AMBER 14SB and AMBER 19SB belong to the same family of force field models, they do differ in the atomic partial charges, the non‐coulombic parameters and the treatment of bonded interactions, foremost the dihedral degrees of freedom. This implies that both the minimum configuration of the residues in the MM zone as well as their interaction with the QM atoms are to some extent different. In order to provide a consistent analysis, this comparison has also been carried out at RIMP2/cc‐pVDZ level. However, due to its increased computational demand the triple‐zeta valence basis set cc‐pVTZ was not considered. In addition, only the GFN2‐xTB method was employed to represent the xTB approach, since the different orders resulted in quite consistent trends (see Figure 4B).

**FIGURE 5:**
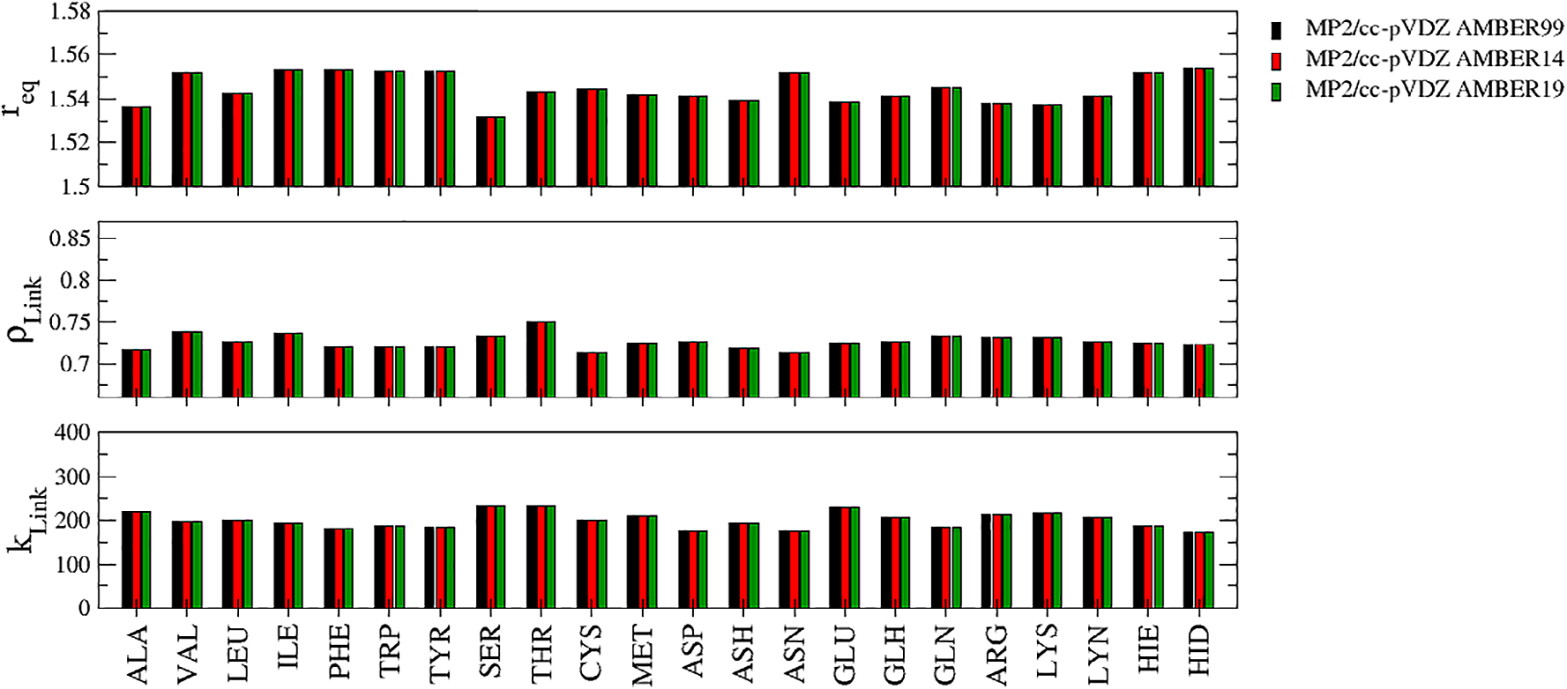
Comparison of the link bond parameters *r*_eq_ in Å, *ρ*_Link_ and *k*_Link_ in kcal mol^−1^ Å^−2^ determined for the 22 considered ACE‐AA‐NME model systems at the RIMP2/cc‐pVDZ level in conjunction with different AMBER force field parametrizations

It can be seen from Figure 5 that despite the differences in the partial charge distributions, the Lennard‐Jones parameters and/or the changed bonded interactions, virtually identical link bond parameters are obtained at the RIMP2/cc‐pVDZ level. Similar trends were observed for all other considered levels in this analysis being SCC DFTB in conjunction with the 3ob and mio parametrization as well as GFN2‐xTB as shown in the Supplementary material Figures S1–S3.

These findings imply that it appears adequate to transfer link bond parameters between different force fields of the same family. Although it would be of particular interest to also assess whether this conclusion still holds true when transferring parameters between different force field families, this step was not considered in the present work. This is due to the fact that related force fields such OPLS‐AA^77^ employ effectively the same potential energy expressions. In this case, it can be expected that a similar finding as for the different AMBER subtypes can be obtained. More difficult, however, is the extension towards other force fields such as CHARMM22^78^ or GROMOS^79^, ^80^ that often employ a united atom (UA) approach combining aliphatic carbon atoms and their bound hydrogen atoms into a single meta‐particle. In this case, additional steps during the QM/MM initialization are required to re‐introduce the missing hydrogen atoms in the QM zone, which make these force field approaches more challenging to implement in the automated link atom parametrization. Moreover, the united atom approach is less effective within the context of an electrostatic embedding framework, which makes UA‐based force fields to some extent less attractive for QM/MM applications compared to their all‐atom counterparts.

### 3.3 Analysis of QM versus QM/MM partial charges

A general question associated to QM/MM‐type simulations is the comparability in the description of the electronic structure in the QM/MM treatment against all‐QM reference data. A particularly useful probe enabling such a comparison are atomic partial charges. While from a physical perspective such partial charges are not non‐observable properties they still provide a mathematical tool to represent local electron density information via single‐atom properties. In this work Mulliken populations^81^ have been employed to evaluate the calculations results obtained at RIMP2/cc‐pVTZ and GFN2‐xTB level, since this framework is available in both associated QM packages Turbomole^65^ and xTB.^55^, ^56^, ^57^ While Mulliken charges have been challenged due to their high sensitivity with respect to the employed level of theory and basis set, they provide an ideal means to compare the all‐QM and QM/MM systems provided all other calculation settings are kept identical.

The different amino acids considered in this study display rather larger variations in the total number of atoms, making a comparison of every single partial charge quite tedious. However, when analyzing the respective data it proofed sufficient to compare only the partial charges of carbon atoms based on the respective location in the individual side chains typically given as C_β_, C_γ_, C_δ_, and so on.

**FIGURE 6:**
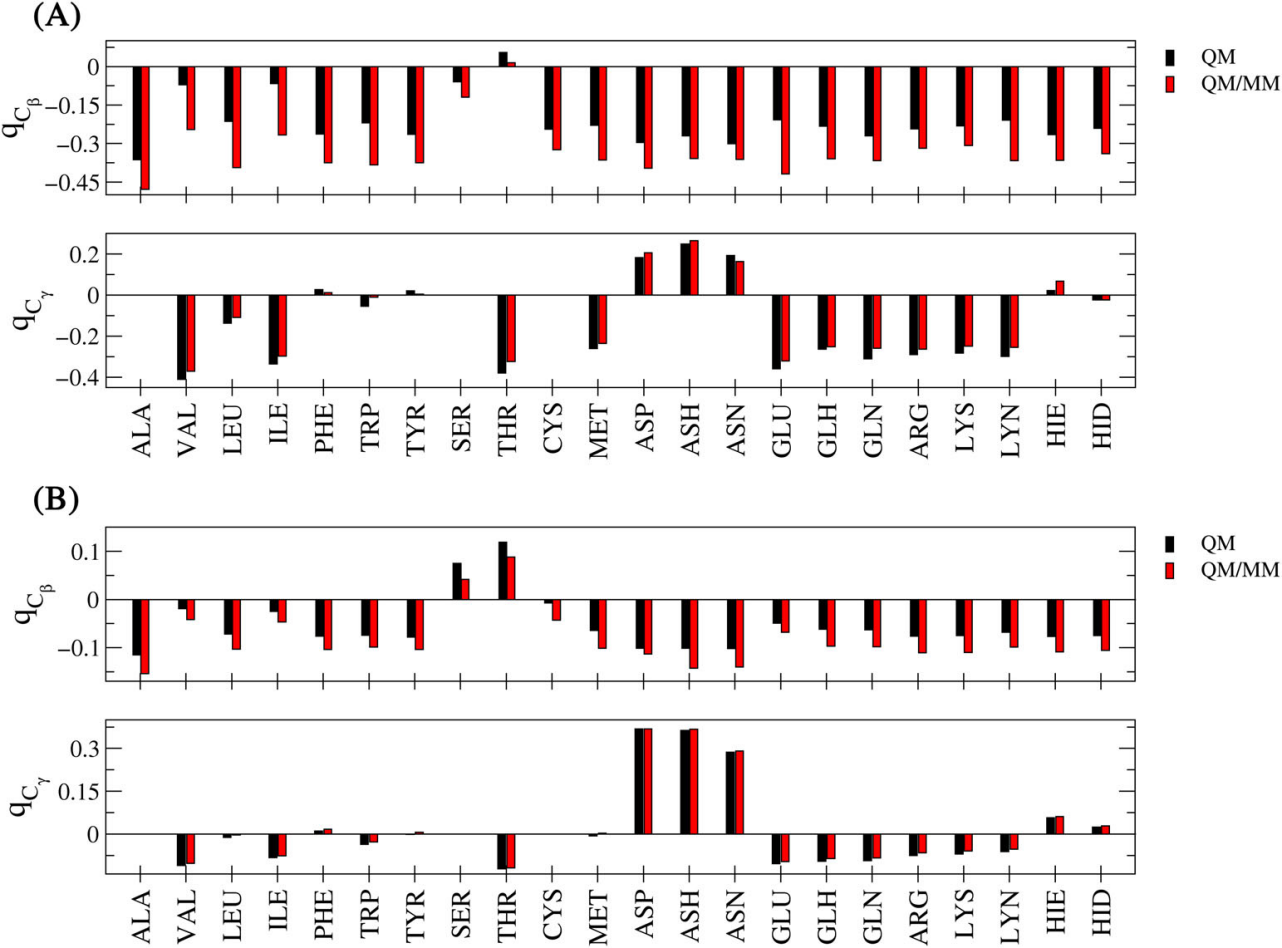
Comparison of the Mulliken partial charges in units of the elementary charge *e* for the C_β_ and C_γ_ atoms obtained from the all‐QM calculation and the QM/MM calculation for the 22 considered ACE‐AA‐NME model systems at (A) the RIMP2/cc‐pVTZ and (B) the GFN2‐xTB level, respectively

Figure 6 depicts the comparison of the Mulliken partial charges for the C_β_ and C_*γ*_ atoms obtained from the all‐QM and QM/MM calculations of the individual amino acids model systems employing the all‐QM optimized configurations. In case more than one carbon atom is found at a given position of a particular side chain, the average value is shown in Figure 6. Not surprisingly, notably differences for partial charges of the C_β_ atoms are observed since they are closest to the QM/MM interface and the link bond. Nevertheless, the overall trends observed in the all‐QM calculations are well‐reflected at QM/MM level in both the RIMP2/cc‐pVTZ and GFN2‐xTB case. The observed deviations are greatly reduced in case of the C_*γ*_ atoms, implying that the description of the electronic structure is largely comparable between the all‐QM and QM/MM case. This trend is continued for other carbon atoms upon increasing distance from the QM/MM interface (see Supplementary material Tables S8 and S9).

These findings indicate that the QM/MM strategy indeed provides a highly suitable approach to describe the electronic structure of the chosen sub‐system with the largest inconsistencies being observed close to the QM/MM interface. In case an accurate description of the C_β_ atoms represent a crucial element in a particular study (e.g., NMR properties), it might prove necessary to consider an overall increase of the QM zone to also encompass atoms of the associated amino acid backbone. This requires the construction of link bond parameters associated to fragmented peptide bonds in the backbone which due to the polar nature is more difficult compared to the treatment of C_α_—C_β_ bonds. Alternatively, the use of a different framework to treat these QM/MM frontier bonds may be considered in this particular case. However, since most of these methods require very specific changes in the employed QM software, the key advantage of the link bond approach requiring no modification of existing QM programs is lost. No general recommendation can be given in this particular case and the best strategy has to be determined for the specific research question at hand.

### 3.4 Impact of non‐ideal link bond settings

In order to demonstrate the benefit in pre‐optimizing the link bond parameters a short exemplary QM/MM MD simulation of a hydrated peptide has been carried out. The comparably short 42 amino acid *β*‐amyloid peptide coordinated to a Zn^2+^ ion derived from pdb‐structure 1ZE9^82^ has been considered. In this example the ion and the directly coordinated side chains of one glutamate and three histidine residues were included in the QM zone treated at GFN2‐xTB level.^56^ The AMBER 14SB force field^49^ in conjunction with the TIP3P water model^83^ has been applied to describe the interactions in the MM region as well as the QM/MM potential coupling. Prior to the QM/MM MD simulation the system has been pre‐equilibrated to standard conditions (i.e., 298.15 K and 1.013 bar) via classical MD for a total of 0.5 ns. Next, a short re‐equilibration 25 ps (12,500 MD steps) has been carried out, followed by 50 ps (25,000 MD steps) of sampling.

**FIGURE 7:**
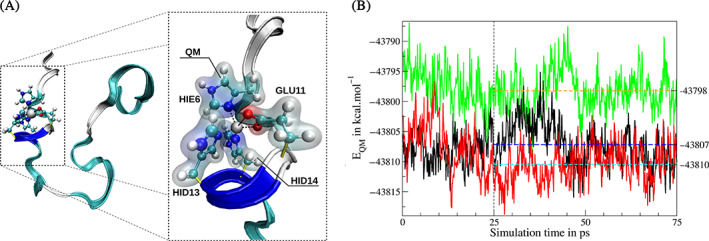
(A) Snapshot of the *β*‐amyloid/Zn^2+^ complex highlighting the employed QM region. (B) Time evolution of the total GFN2‐xTB energies obtained from the 75 ps simulations of the *β*‐amyloid/Zn^2+^ system employing the ideal link atom ratio *ρ*_*Link*_ (black) as well as non‐ideal settings with *ρ*_*Link*_ being increased (red) or decreased (green) by 0.1 for all involved QM/MM frontier bonds. The respective average values indicated by the dashed lines have been determined excluding the initial equilibration phase of 25 ps

In addition to executing a short proof‐of‐concept QM/MM MD simulation employing the ideal link bond parameters derived in this work, two additional simulations have been carried out, thereby deliberately using non‐ideal settings for the link‐bond ratio *ρ*_*Link*_ changed by ±0.1 for all involved QM/MM link bonds. All other associated parameters, namely the equilibrium distances *r*_*eq*_ and the adjusted bond force constants *k*_*Link*_, remained unmodified in this test. All three simulations have been started from the same pre‐equilibrated structure shown in Figure 7A.

Despite the fact that the simulation time of 75 ps is much to short to enable a detailed investigation of the ion‐peptide complex and its influence on the overall peptide structure, it is sufficient to demonstrate the impact of ill‐defined link bond parameters. Although the three different simulations have been started from the same initial structure the time evolution of the QM energy quickly diverges (see Figure 7B). While this can to some extend be expected due to the deterministic nature of MD simulations, the average values observed for the last 50 ps of the simulation time clearly demonstrate that the use of unoptimized link bond settings have the potential to influence the total QM energy in a negative way, especially when too large values for *ρ*_*Link*_ are employed. Since QM‐derived observables are either directly or indirectly dependent on the description of the QM energy, the use of adjusted link bond settings is strongly recommended. Otherwise the calculated energetic data as well as derived properties such as atomic forces may be subject to errors with an unknown magnitude, which ultimately may lead to wrong conclusions about the simulation system.

**FIGURE 8:**
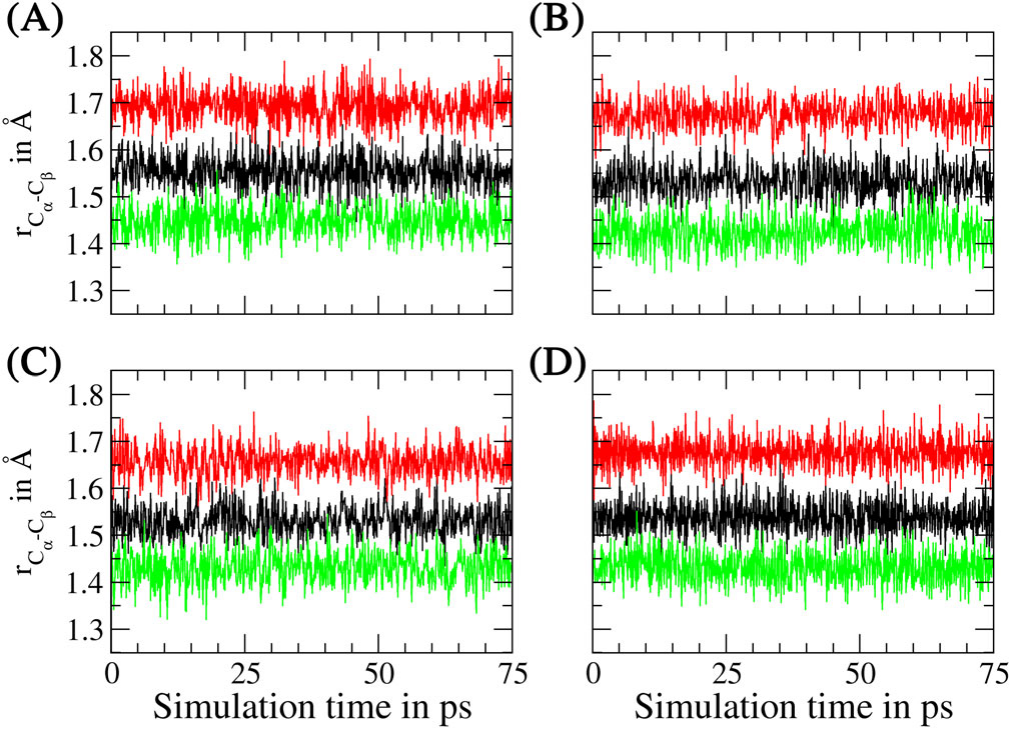
Comparison of the C_α_—C_β_ bond distance of the four amino acid residues (A) GLU‐11, (B) HIE‐6, (C) HID‐13, and (D) HIE‐14 along the 75 ps GFN2‐xTB/MM MD simulations of the *β*‐amyloid/Zn^2+^ complex employing the ideal distance ratios *ρ*_*Link*_ (black) as well as non‐ideal settings corresponding to an increase (red) and decrease (green) of *ρ*_*Link*_ by 0.1 for all involved QM/MM frontier bonds

Within this short test simulations the structural properties close to the center of the QM zone are not influenced to a large extend by the non‐ideal link atom placement, resulting in deviations in the range of 1–2% from the ideal case (data not shown). On the other hand, the average distances observed for the C_α_—C_β_ bonds of the four coordinating amino acids shown in Figure 8 suffer greatly when non‐ideal distance ratios are applied. A misplacement of the hydrogen atoms employed to saturate the QM/MM frontier bonds by just *±*0.1 in the applied distance ratio ρ*_Link_* results in a significant increase/decrease of the bond length in the range of 0.1–0.15 Å, whereas the ideal ratio yields bond distances close to the ideal value determined for the small model systems employed in this study. Since these deviations from the ideal setup are persistent over the entire course of a QM/MM MD study, it can be expected that the associated errors accumulate over the simulation period. This may not only affect the description inside the QM region as seen from the respective energy time series but also negatively influence MM residues in close vicinity of the link bonds. In the worst case scenario the associated errors may then even propagate towards all regions in the simulation system via collision events with other amino acid side chains or/and solvent molecules.

## 4 CONCLUSION

The parametrization of ideal link bond parameters for five different tight binding based semi‐empirical QM methods carried out in this work provides a valuable resource for the execution of QM/MM simulations of biomacromolecular systems. The comparison of the three link bond parameters {*r*_*eq*_, *ρ*_*Link*_, *k*_*Link*_} among the different methods as well as with high‐level reference data obtained at the RIMP2/cc‐pVXZ (X = D,T) level of theory provides a clear indication that structure‐ and energy‐adjusted QM/MM link bonds are preferred over the use of a single global link bond setting. The latter is true when comparing the different amino acid residues as well as when considering different quantum chemical calculation methods. As a consequence, the transfer of link bond parameters calculated using a particular high‐level reference method such as RIMP2 to lower levels of theory such as DFTB is not a recommended course of action. On the contrary, a particular QM/MM study will strongly benefit from optimizing the link bond parameters for the individual amino acids in question employing the outlined procedure prior to the execution of the actual QM/MM study. Thus, the reported link atom parameters determined in this work provide a valuable primer for QM/MM simulations employing the considered tight binding methods or provide a starting point for the re‐parametrization in the respective chemical environment.

In contrast to the limited transferability of *r*_*eq*_, *ρ*_*Link*_ and *k*_*Link*_ between different amino acids and QM methods, the comparison of the link bond parameters determined for different flavors of the AMBER force field showed that effectively identical parameterizations are obtained. This implies that a transfer of the link bond settings between different force fields appears viable, although an additional evaluation employing different force field families should be conducted to further investigate this aspect.

The comparison of atomic partial charges obtained at all‐QM and QM/MM level carried out for two representative levels of theory (RIMP2/cc‐pVTZ, GFN2‐xTB) clearly points out that the largest deviation in the description of the electronic structure is observed for atoms close to the QM/MM frontier bonds. However, the agreement between the partial charge data was shown to progressively improve upon increasing distance from the link bond as expected.

The impact of non‐optimized link bond settings could be demonstrated in a short QM/MM MD simulation of the hydrated *β*‐amyloid/Zn^2+^ complex, including the ion and its four coordinating amino acid side chains into the QM treatment. Strong deviations in the average bond distance of the associated C_α_—C_β_ bonds along with a notable impact onto the total QM energy have been observed when employing non‐ideal the link bond settings.

The data summarized in this work is the result of an extensive probing of the link bond properties of the investigated amino acid model systems based on an exhaustive number of individual QM/MM C_α_—C_β_ bond scans. Considering the increasing success of tight binding based approaches resulting from their exceptional accuracy/effort ratio (when remaining within the scope of the associated DFTB parametrization), it can be expected that QM/MM‐type simulations of biomolecular systems in conjunction with DFTB methods will become increasingly attractive already in the near future.

## Supporting information


**Tables S1–S7**: Link bond parameters for the seven different levels of theory being RIMP2/cc‐pVXZ (X = D,T), SCC DFTB/3ob, SCC DFTB/mio as well as GFN*n*‐xTB (*n* = 0, 1, 2) in conjunction with the AMBER 99SB, AMBER 14SB and AMBER 19SB force filed parametrization, respectively.
Tables S8–S9: Comparison of Mulliken partial charges *q* of the side chain carbon atoms for each of the 22 amino acids obtained at the RIMP2/cc‐pVTZ and GFN2‐xTB level of theory.
Figures S1–S3: Comparison of link bond parameters obtained for RIMP2/cc‐pVDZ, SCC DFTB/3ob, SCC DFTB/mio and GFN2‐xTB in conjunction with the AMBER 99SB, AMBER 14SB and AMBER 19SB, respectively.

## Data Availability

The data that supports the findings of this study are available in the supplementary material of this article.
