# Supplementary material for: Probing the range of applicability of structure‐ and energy‐adjusted QM/MM link bonds II: Optimized link bond parameters for density functional tight binding approaches
Source: J Comput Chem. 2022 Mar 3;43(11):746–56. doi: 10.1002/jcc.26830 (PMC9314059; doi:10.1002/jcc.26830)
Supplement: Supplementary file 1 — Tables S1–S7: Link bond parameters for the seven different levels of theory being RIMP2/cc‐pVXZ (X = D,T), SCC DFTB/3ob, SCC DFTB/mio as well as GFNn‐xTB (n = 0, 1, 2) in conjunction with the AMBER 99SB, AMBER 14SB and AMBER 19SB force filed parametrization, respectively. Tables S8–S9: Comparison of Mulliken partial charges q of the side chain carbon atoms for each of the 22 amino acids obtained at the RIMP2/cc‐pVTZ and GFN2‐xTB level of theory. Figures S1–S3: Comparison of link bond parameters obtained for RIMP2/cc‐pVDZ, SCC DFTB/3ob, SCC DFTB/mio and GFN2‐xTB in conjunction with the AMBER 99SB, AMBER 14SB and AMBER 19SB, respectively. [file JCC-43-746-s001.pdf]

Probing the Range of Applicability of Structure- and Energy-adjusted QM/MM Link Bonds II – Optimized Link Bond Parameters for Tight Binding Approaches

Supplementary Material

*H. Georg Gallmetzer and Thomas S. Hofer**

Theoretical Chemistry Division
Institute of General, Inorganic and Theoretical Chemistry
Center for Chemistry and Biomedicine
University of Innsbruck, Innrain 80-82, A-6020 Innsbruck, Austria
E-Mail: T.Hofer@uibk.ac.at
Tel.: +43-512-507-57111
Fax: +43-512-507-57199

February 28, 2022

*Corresponding author

S.I Optimized QM/MM link bond parameters

In this section the optimized link bond parameters $\{\rho, \rho_{Link}, k_{Link}\}$ obtained at different levels of theory as outlined in the main article are listed. The ideal link bond ratio ρ_{Link} is dimensionless.

Table S1: Link bond parameters r_{eq} in Å, ρ_{Link} and k_{Link} in kcal.mol^{-1}Å^{-2}) btained for the different amino acid residues at RIMP2/cc-pVTZ level.

	AMBER 19SB		
	r_{eq}	ρ_{Link}	k_{Link}
ALA	1.5294395	0.70976580	203.8104
VAL	1.5447512	0.73249255	185.3463
LEU	1.5352222	0.71965140	184.4616
ILE	1.5454708	0.73218975	177.5868
PHE	1.5456324	0.71307157	165.5441
TRP	1.5448609	0.71253520	167.5493
TYR	1.5452601	0.71379936	167.2332
SER	1.5246928	0.72540565	215.0416
THR	1.5365041	0.74302906	210.6280
CYS	1.5371561	0.70824482	182.5618
MET	1.5349398	0.71859724	194.8085
ASP	0.5331476	0.72322899	170.2340
ASH	1.5314943	0.71468942	182.0576
ASN	1.5453798	0.70777666	160.0960
GLU	1.5292698	0.72170708	227.0630
GLH	1.5334730	0.72005521	196.7724
GLN	1.5365466	0.72754781	175.9817
ARG	1.5247985	0.72501708	188.2586
LYS	1.5277918	0.72234922	206.1127
LYN	1.5331206	0.71794512	194.1389
HIE	1.5450881	0.71597185	160.8581
HID	1.5468251	0.71713984	157.7357

Table S2: Link bond parameters r_{eq} in Å, ρ_{Link} and k_{Link} in kcal.mol^{-1}Å^{-2}) obtained for the different amino acid residues at RIMP2/cc-pVDZ level.

	AMBER 99SB			AMBER 14SB			AMBER 19SB		
	r_{eq}	ρ_{Link}	k_{Link}	r_{eq}	ρ_{Link}	k_{Link}	r_{eq}	ρ_{Link}	k_{Link}
ALA	1.5360026	0.71619586	218.6720	1.5360026	0.71619586	218.6719	1.5360026	0.71619586	218.6719
VAL	1.5521840	0.73764254	197.0090	1.5521840	0.73764254	197.0088	1.5521840	0.73764254	197.0090
LEU	1.5426216	0.72730349	199.3978	1.5426216	0.72730349	199.3978	1.5426216	0.72730349	199.3978
ILE	1.5531486	0.73725184	193.7869	1.5531486	0.73725184	193.7871	1.5531486	0.73725184	193.7869
PHE	1.5529251	0.72003677	182.0630	1.5529251	0.72003678	182.0630	1.5529251	0.72003678	182.0628
TRP	1.5528783	0.71976429	188.0749	1.5528783	0.71976325	188.0660	1.5528783	0.71976427	188.0756
TYR	1.5526106	0.72020841	185.6400	1.5526106	0.72020841	185.6400	1.5526106	0.72020842	185.6406
SER	1.5313773	0.73306263	232.2417	1.5313773	0.73306263	232.2417	1.5313773	0.73306263	232.2417
THR	1.5429132	0.74975101	232.0096	1.5429132	0.74975101	232.0096	1.5429132	0.74975101	232.0096
CYS	1.5445654	0.71381884	201.6555	1.5445654	0.71381884	201.6556	1.5445654	0.71370291	199.5056
MET	1.5419993	0.72542809	209.1979	1.5419993	0.72542808	209.1990	1.5419993	0.72540384	209.4290
ASP	1.5414329	0.72718947	176.3857	1.5414329	0.72718947	176.3855	1.5414329	0.72718947	176.3855
ASH	1.5394632	0.71758779	194.3190	1.5394632	0.71758779	194.3190	1.5394632	0.71758779	194.3190
ASN	1.5521624	0.71362153	174.1219	1.5521624	0.71362153	174.1219	1.5521624	0.71362153	174.1220
GLU	1.5388062	0.72431617	228.0010	1.5388062	0.72431617	228.0010	1.5388062	0.72431617	228.0011
GLH	1.5410146	0.72617126	206.1167	1.5410146	0.72617126	206.1166	1.5410146	0.72617126	206.1165
GLN	1.5448518	0.73282417	183.5050	1.5448518	0.73282417	183.5052	1.5448518	0.73282417	183.5051
ARG	1.5382275	0.73083881	213.0698	1.5382275	0.73083881	213.0698	1.5382275	0.73083881	213.0698
LYS	1.5374792	0.73191453	215.2882	1.5374792	0.73191453	215.2883	1.5374792	0.73191453	215.2881
LYN	1.5412545	0.72621195	206.4551	1.5412545	0.72621195	206.4549	1.5412545	0.72621195	206.4549
HIE	1.5517955	0.72433399	189.0738	1.5517955	0.72433399	189.0740	1.5517955	0.72433399	189.0738
HID	1.5539933	0.72284546	172.2608	1.5539933	0.72284546	172.2608	1.5539933	0.72284546	172.2608

Table S3: Link bond parameters r_{eq} in Å, ρ_{Link} and k_{Link} in kcal.mol^{-1}Å^{-2}) obtained for the different amino acid residues at SCC DFTB/3ob level.

	AMBER 99SB			AMBER 14SB			AMBER 19SB		
	r_{eq}	ρ_{Link}	k_{Link}	r_{eq}	ρ_{Link}	k_{Link}	r_{eq}	ρ_{Link}	k_{Link}
ALA	1.5411055	0.70497107	182.2486	1.5411032	0.70496804	182.2497	1.5411059	0.70496822	182.2523
VAL	1.5729635	0.72542897	66.8772	1.5729659	0.72542695	66.8961	1.5729647	0.72542758	66.8933
LEU	1.5572155	0.71282995	108.8533	1.5572120	0.71283386	108.8575	1.5572127	0.71282794	108.8701
ILE	1.5730506	0.72535269	63.1268	1.5730455	0.72536030	63.1227	1.5730502	0.72537199	63.0888
PHE	1.5656942	0.70976114	96.5686	1.5656868	0.70976086	96.5916	1.5656897	0.70976682	96.5735
TRP	1.5656324	0.70700328	108.6533	1.5656338	0.70700901	108.6471	1.5656308	0.70701993	108.6249
TYR	1.5654658	0.70972939	98.0966	1.5654694	0.70973016	98.0774	1.5654752	0.70973266	98.0269
SER	1.5466224	0.72051479	145.1815	1.5466212	0.72051967	145.1730	1.5466220	0.72052031	145.1597
THR	1.5767976	0.74794158	22.2571	1.5768088	0.74794977	22.1958	1.5768055	0.74806805	22.2731
CYS	1.5499377	0.70997735	136.5611	1.5499353	0.70997883	136.5590	1.5499344	0.70990530	136.6166
MET	1.5595774	0.71257057	113.2362	1.5595735	0.71256428	113.2619	1.5595729	0.71254940	113.2867
ASP	1.5508750	0.69790028	159.1571	1.5508757	0.69789946	159.1431	1.5508738	0.69790204	159.1569
ASH	1.5576187	0.71906417	81.1624	1.5576173	0.71910687	81.0862	1.5576167	0.71909206	81.1262
ASN	1.5656621	0.69750697	112.5496	1.5656609	0.69750804	112.5550	1.5656593	0.69750649	112.5594
GLU	1.5552750	0.71143388	139.9523	1.5552848	0.71143069	139.9241	1.5552766	0.71143363	139.9390
GLH	1.5586280	0.71509562	101.2581	1.5586316	0.71509497	101.2532	1.5586289	0.71509386	101.2395
GLN	1.5605454	0.72379037	65.6806	1.5605296	0.72377421	65.7927	1.5605492	0.72384673	65.5947
ARG	1.5580604	0.71429409	100.1118	1.5580694	0.71429004	100.0924	1.5580681	0.71430822	100.0582
LYS	1.5582864	0.71476258	97.4658	1.5582848	0.71476595	97.4415	1.5582716	0.71474775	97.5477
LYN	1.5568623	0.71215084	117.3342	1.5568611	0.71215688	117.3254	1.5568599	0.71214893	117.3521
HIE	1.5678161	0.70238067	121.7261	1.5678179	0.70237493	121.7501	1.5678233	0.70237005	121.7566
HID	1.5707598	0.71015880	76.8716	1.5707489	0.71016368	76.8666	1.5707563	0.71015849	76.8694

Table S4: Link bond parameters r_{eq} in Å, ρ_{Link} and k_{Link} in kcal.mol^{-1}Å^{-2}) obtained for the different amino acid residues at SCC DFTB/mio level.

	AMBER 99SB			AMBER 14SB			AMBER 19SB		
	r_{eq}	ρ_{Link}	k_{Link}	r_{eq}	ρ_{Link}	k_{Link}	r_{eq}	ρ_{Link}	k_{Link}
ALA	1.5231947	0.72136816	337.4649	1.5231934	0.72138918	337.4892	1.5231942	0.72136349	337.4566
VAL	1.5467163	0.77471466	250.7242	1.5467164	0.77470465	250.7242	1.5467177	0.77470030	250.7145
LEU	1.5353112	0.74692486	272.7732	1.5353121	0.74692400	272.7677	1.5353133	0.74696318	272.7436
ILE	1.5469062	0.77406015	247.4897	1.5469069	0.77406920	247.4887	1.5469046	0.77406695	247.5112
PHE	1.5410855	0.74235636	255.9235	1.5410889	0.74236481	255.8944	1.5410885	0.74233848	255.9258
TRP	1.5411631	0.73647378	261.3106	1.5411622	0.73644327	261.2755	1.5411587	0.73642542	261.2819
TYR	1.5407571	0.74086127	258.6297	1.5407547	0.74077025	258.5639	1.5407550	0.74082326	258.5992
SER	1.5255413	0.74408943	349.1596	1.5255403	0.74408687	349.1481	1.5255396	0.74408011	349.1395
THR	1.5401299	0.81257637	261.0666	1.5401268	0.81252219	261.1158	1.5401271	0.81255725	261.0849
CYS	1.5356213	0.81090475	253.9914	1.5356211	0.81093641	253.9848	1.5356209	0.81100040	253.2571
MET	1.5372841	0.74279965	274.2622	1.5372858	0.74281242	274.2493	1.5372835	0.74277200	274.2210
ASP	1.5276795	0.73871316	362.5802	1.5276798	0.73871532	362.5943	1.5276851	0.73871082	362.4942
ASH	1.5340375	0.77180789	285.3771	1.5340363	0.77180477	285.3872	1.5340361	0.77183985	285.4332
ASN	1.5411352	0.72346982	241.9165	1.5411332	0.72346721	241.9381	1.5411357	0.72347126	241.9315
GLU	1.5323282	0.73113048	320.5704	1.5323279	0.73113910	320.5737	1.5323240	0.73113092	320.5923
GLH	1.5360074	0.74941044	271.5452	1.5360085	0.74942061	271.5150	1.5360076	0.74940412	271.5172
GLN	1.5392653	0.71994446	276.0024	1.5393652	0.71994929	275.9105	1.5393077	0.71991272	275.8352
ARG	1.5363897	0.75972666	270.4586	1.5363866	0.75973926	270.4648	1.5363865	0.75973378	270.4749
LYS	1.5363749	0.75927694	270.0403	1.5363793	0.75926696	270.0319	1.5363765	0.75924782	270.0139
LYN	1.5349280	0.74239578	282.4540	1.5349294	0.74240512	282.4820	1.5349288	0.74240270	282.4638
HIE	1.5446206	0.72434361	351.3623	1.5446236	0.72434347	351.3367	1.5446236	0.72434252	351.3635
HID	1.5442291	0.74365749	233.7212	1.5442375	0.74364648	233.6382	1.5442260	0.74362498	233.6916

Table S5: Link bond parameters r_{eq} in Å, ρ_{Link} and k_{Link} in kcal.mol^{-1}Å^{-2}) obtained for the different amino acid residues at GFN0-xTB level.

	AMBER 99SB			AMBER 14SB			AMBER 19SB		
	r_{eq}	ρ_{Link}	k_{Link}	r_{eq}	ρ_{Link}	k_{Link}	r_{eq}	ρ_{Link}	k_{Link}
ALA	1.5237555	0.71402025	271.6656	1.5234773	0.72645095	197.3252	1.5234773	0.72645095	197.3252
VAL	1.5357911	0.75485115	153.0322	1.5363135	0.78547929	190.8232	1.5363135	0.78547929	190.8230
LEU	1.5324644	0.72964353	121.8999	1.5322313	0.74656907	168.5139	1.5322313	0.74656907	168.5139
ILE	1.5360741	0.76027093	161.6160	1.5373069	0.78346481	184.3930	1.5373069	0.78346481	184.3930
PHE	1.5378638	0.72904638	228.5988	1.5281729	0.75762818	199.5897	1.5281729	0.75762818	199.5895
TRP	1.5394020	0.73349726	279.0739	1.5252446	0.74840552	204.3097	1.5252446	0.74840552	204.3097
TYR	1.5384380	0.72866441	226.0307	1.5315813	0.75008250	190.8730	1.5315813	0.75008250	190.8728
SER	1.5232220	0.74609488	267.1871	1.5242471	0.76998517	198.6394	1.5242471	0.76998517	198.6394
THR	1.5280399	0.75976784	220.2109	1.5286906	0.78344315	177.4488	1.5286906	0.78344315	177.4489
CYS	1.5291575	0.72244970	250.9655	1.5253754	0.74195501	206.9801	1.5253754	0.74172252	206.6210
MET	1.5303018	0.72262480	241.7673	1.5320041	0.74292962	167.8736	1.5320041	0.74290580	167.8375
ASP	1.5291060	0.80935385	208.2598	1.5315448	0.86054587	207.8188	1.5315448	0.86054588	207.8224
ASH	1.5315691	0.76753525	99.3893	1.5300622	0.75834443	191.5512	1.5300622	0.75834443	191.5511
ASN	1.5407203	0.82488424	172.3757	1.5320784	0.73612644	152.4621	1.5320784	0.73612644	152.4621
GLU	1.5311767	0.73777725	84.3284	1.5325979	0.75859108	166.1164	1.5325979	0.75859108	166.1163
GLH	1.5298153	0.73900308	253.2762	1.5308750	0.75437740	162.9933	1.5308750	0.75437740	162.9935
GLN	1.5280314	0.72789526	250.3156	1.5291167	0.75200391	167.9681	1.5291167	0.75200391	167.9683
ARG	1.5293406	0.72387626	105.7679	1.5297917	0.73748978	172.8326	1.5297917	0.73748978	172.8326
LYS	1.5298920	0.72191686	261.5535	1.5311560	0.73709926	173.8094	1.5311560	0.73709926	173.8094
LYN	1.5299937	0.72867941	251.0583	1.5312767	0.75009942	167.0782	1.5312767	0.75009942	167.0783
HIE	1.5388649	0.72856086	62.2516	1.5342222	0.74343302	182.0713	1.5342222	0.74343302	182.0712
HID	1.5382832	0.73104219	257.7037	1.5322550	0.74873151	181.6103	1.5322550	0.74873151	181.6103

Table S6: Link bond parameters r_{eq} in Å, ρ_{Link} and k_{Link} in kcal.mol^{-1}Å^{-2}) obtained for the different amino acid residues at GFN1-xTB level.

	AMBER 99SB			AMBER 14SB			AMBER 19SB		
	r_{eq}	ρ_{Link}	k_{Link}	r_{eq}	ρ_{Link}	k_{Link}	r_{eq}	ρ_{Link}	k_{Link}
ALA	1.5338975	0.71745714	245.7543	1.5338975	0.71745714	245.7543	1.5338975	0.71745714	245.7543
VAL	1.5505222	0.74771236	205.6425	1.5505222	0.74771236	205.6426	1.5505222	0.74771236	205.6426
LEU	1.5438149	0.73369413	218.3434	1.5438149	0.73369412	218.3434	1.5438149	0.73369412	218.3434
ILE	1.5532825	0.75375571	201.7768	1.5532825	0.75375571	201.7768	1.5532825	0.75375572	201.7775
PHE	1.5416930	0.73960506	242.2331	1.5416930	0.73960506	242.2332	1.5416930	0.73960506	242.2333
TRP	1.5354850	0.74176200	263.1980	1.5354850	0.74176200	263.1980	1.5354850	0.74176200	263.1980
TYR	1.5406470	0.73936713	247.4759	1.5406470	0.73936713	247.4759	1.5406470	0.73936713	247.4759
SER	1.5403341	0.74403100	253.3272	1.5403341	0.74403100	253.3272	1.5403341	0.74403100	253.3272
THR	1.5481339	0.74204836	201.7244	1.5481339	0.74204836	201.7244	1.5481339	0.74204836	201.7246
CYS	1.5364724	0.72779858	247.6607	1.5364724	0.72779858	247.6608	1.5364724	0.72761174	247.4083
MET	1.5445377	0.72691021	220.9086	1.5445377	0.72691021	220.9085	1.5445377	0.72689261	220.8947
ASP	1.5362383	0.80423811	226.5238	1.5362383	0.80423811	226.5238	1.5362383	0.80423811	226.5238
ASH	1.5411482	0.74194477	231.3889	1.5411482	0.74194477	231.3888	1.5411482	0.74194477	231.3889
ASN	1.5426352	0.74318712	245.5289	1.5426352	0.74318712	245.5291	1.5426352	0.74318712	245.5289
GLU	1.5523123	0.72748929	206.4190	1.5523123	0.72748929	206.4191	1.5523123	0.72748929	206.4192
GLH	1.5451702	0.73033459	212.4518	1.5451702	0.73033459	212.4520	1.5451702	0.73033459	212.4518
GLN	1.5401438	0.72611757	220.3346	1.5401438	0.72611757	220.3346	1.5401438	0.72611757	220.3346
ARG	1.5460307	0.74048955	212.1993	1.5460307	0.74048956	212.1992	1.5460307	0.74048955	212.1993
LYS	1.5449029	0.74317805	142.8466	1.5449029	0.74317805	142.8468	1.5449029	0.74317805	142.8467
LYN	1.5446849	0.73420726	213.6450	1.5446849	0.73420726	213.6450	1.5446849	0.73420726	213.6451
HIE	1.5430710	0.73082607	233.4955	1.5430710	0.73082607	233.4955	1.5430710	0.73082607	233.4955
HID	1.5444727	0.73159907	231.8764	1.5444727	0.73159907	231.8764	1.5444727	0.73159907	231.8762

Table S7: Link bond parameters r_{eq} in Å, ρ_{Link} and k_{Link} in kcal.mol^{-1}Å^{-2}) obtained for the different amino acid residues at GFN2-xTB level.

	AMBER 99SB			AMBER 14SB			AMBER 19SB		
	r_{eq}	ρ_{Link}	k_{Link}	r_{eq}	ρ_{Link}	k_{Link}	r_{eq}	ρ_{Link}	k_{Link}
ALA	1.5355497	0.71248475	255.8890	1.5355497	0.71248475	255.8891	1.5355497	0.71248475	255.8891
VAL	1.5523984	0.74690867	232.1409	1.5523984	0.74690868	232.1424	1.5523984	0.74690867	232.1411
LEU	1.5428915	0.72289301	239.3360	1.5428915	0.72289301	239.3360	1.5428915	0.72289301	239.3360
ILE	1.5522969	0.74318005	201.4116	1.5522969	0.74318005	201.4117	1.5522969	0.74318005	201.4115
PHE	1.5400312	0.73514778	258.3213	1.5400312	0.73514778	258.3213	1.5400312	0.73514778	258.3214
TRP	1.5360204	0.73795173	275.4450	1.5360204	0.73795173	275.4449	1.5360204	0.73795173	275.4449
TYR	1.5415654	0.73568224	258.0357	1.5415654	0.73568224	258.0357	1.5415654	0.73568224	258.0355
SER	1.5401174	0.73494772	256.1616	1.5401174	0.73494772	256.1616	1.5401174	0.73494772	256.1616
THR	1.5503496	0.74156987	229.7272	1.5503496	0.74156986	229.7283	1.5503496	0.74156986	229.7284
CYS	1.5340317	0.72110948	242.2948	1.5340317	0.72110949	242.2969	1.5340317	0.72097923	242.0470
MET	1.5417635	0.71844857	125.7223	1.5417635	0.71844857	125.7222	1.5417635	0.71842785	127.0052
ASP	1.5337604	0.78506902	179.2567	1.5337604	0.78506902	179.2567	1.5337604	0.78506902	179.2567
ASH	1.5458323	0.72523049	232.7875	1.5458323	0.72523049	232.7875	1.5458323	0.72523049	232.7876
ASN	1.5378410	0.73163281	124.3648	1.5378410	0.73163281	124.3649	1.5378410	0.73163281	124.3649
GLU	1.5554224	0.72099689	216.7521	1.5554224	0.72099689	216.7521	1.5554224	0.72099689	216.7521
GLH	1.5443519	0.72835252	133.7850	1.5443519	0.72835252	133.7850	1.5443519	0.72835252	133.7850
GLN	1.5423946	0.72386996	238.9337	1.5423946	0.72386996	238.9336	1.5423946	0.72386995	238.9343
ARG	1.5445317	0.73466328	227.0361	1.5445317	0.73466328	227.0362	1.5445317	0.73466328	227.0362
LYS	1.5438098	0.73425746	224.9224	1.5438098	0.73425746	224.9224	1.5438098	0.73425746	224.9222
LYN	1.5432987	0.72437123	239.4482	1.5432987	0.72437123	239.4482	1.5432987	0.72437123	239.4484
HIE	1.5423786	0.72616759	211.7480	1.5423786	0.72616759	211.7479	1.5423786	0.72616759	211.7479
HID	1.5424604	0.73064688	250.1823	1.5424604	0.73064688	250.1824	1.5424604	0.73064688	250.1824

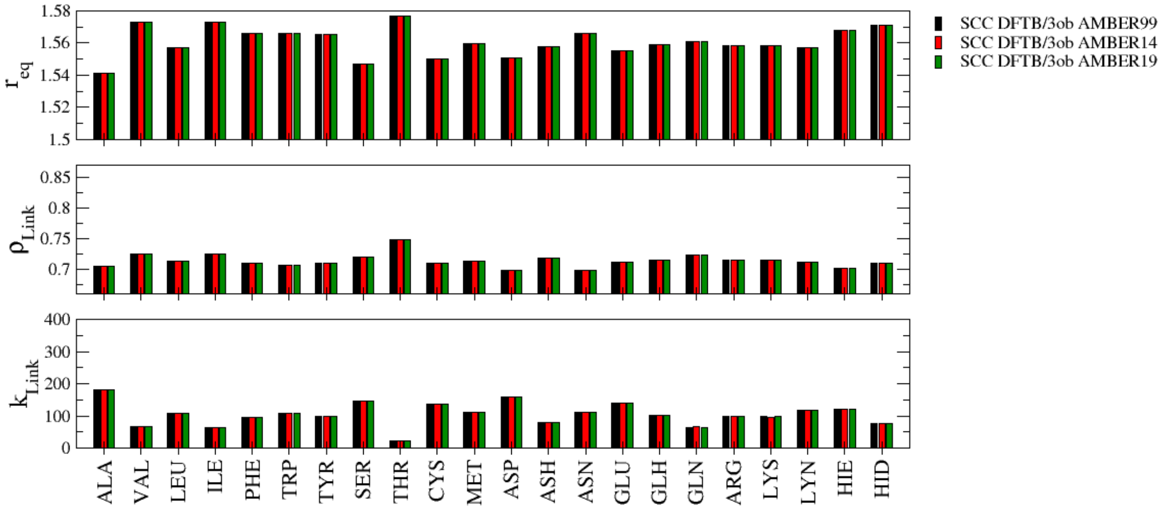

Figure S1: Comparison of the link bond parameters r_{eq} in Å, ρ_{Link} and k_{Link} in kcal.mol^{-1}Å^{-2} determined for the 22 considered ACE-AA-NME model systems at the SCC DFTB/3ob level in conjunction with different AMBER force field parametrization.

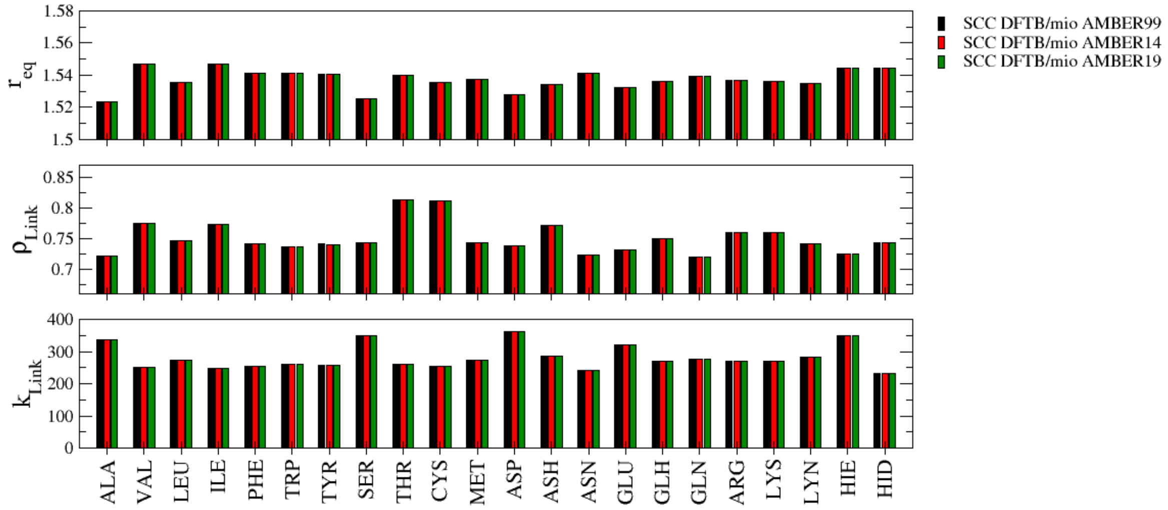

Figure S2: Comparison of the link bond parameters r_{eq} in Å, ρ_{Link} and k_{Link} in kcal.mol^{-1}Å^{-2} determined for the 22 considered ACE-AA-NME model systems at the SCC DFTB/mio level in conjunction with different AMBER force field parametrization.

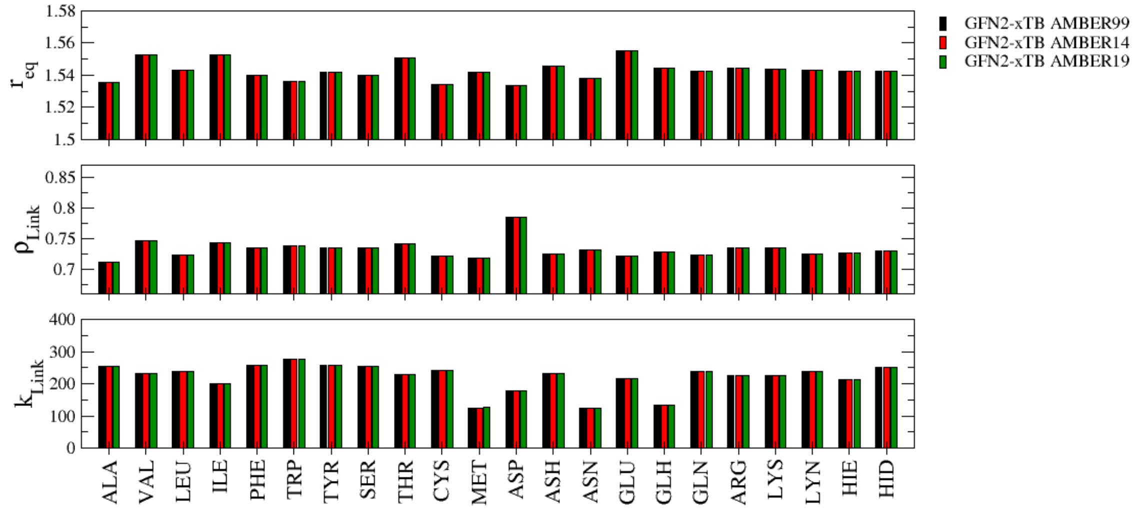

Figure S3: Comparison of the link bond parameters r_{eq} in Å, ρ_{Link} and k_{Link} in kcal.mol^{-1}Å^{-2} determined for the 22 considered ACE-AA-NME model systems at the GFN2-xTB level in conjunction with different AMBER force field parametrization.

S.II QM vs QM/MM partial charge analysis

In this section a comparison of the Mulliken partial charges of all carbon atoms of the respective amino acid (AA) side chains obtained at RIMP2/cc-pVTZ and GFN2-xTB level are compared, enabling to judge the impact of the QM/MM interface on the description of the electronic structure in the respective QM subsystem. Due to their vicinity to the QM/MM boundary and the hydrogen link-atom, the largest differences are observed for the C_β atoms as expected. However, the data shows that the deviations are progressively decreasing upon increasing distance from the QM/MM interface.

Table S8: Mulliken partial charges q in units of the elementary charge e of side chain carbon atoms for each of the 22 amino acids obtained at the RIMP2/cc-pVTZ level. In case more than one carbon atom is present at a given position of a particular AA side chain, the partial charges are provided for the individual atoms.

		C_β	C_γ	C_δ	C_ϵ	C_ζ	C_η
ALA	QM	-0.363					
	QM/MM	-0.478					
VAL	QM	-0.071	-0.385/-0.436				
	QM/MM	-0.245	-0.337/-0.405				
LEU	QM	-0.213	-0.137	-0.391/-0.387			
	QM/MM	-0.394	-0.109	-0.363/-0.377			
ILE	QM	-0.067	-0.399/-0.271	-0.400			
	QM/MM	-0.267	-0.350/-0.243	-0.384			
PHE	QM	-0.263	0.026	-0.180/-0.206	-0.152/-0.156	-0.163	
	QM/MM	-0.375	0.012	-0.159/-0.201	-0.152/-0.147	-0.166	
TRP	QM	-0.220	-0.055	-0.263/0.060	-0.014/-0.123	-0.238/-0.190	-0.153
	QM/MM	-0.383	-0.011	-0.225/0.029	0.013/-0.168	-0.236/-0.174	-0.155
TYR	QM	-0.264	0.020	-0.188/-0.200	-0.244/-0.194	0.217	
	QM/MM	-0.375	0.005	-0.167/-0.194	-0.243/-0.184	0.212	
SER	QM	-0.060					
	QM/MM	-0.119					
THR	QM	0.055	-0.380				
	QM/MM	0.015	-0.324				
CYS	QM	-0.244					
	QM/MM	-0.324					
MET	QM	-0.229	-0.260		-0.365		
	QM/MM	-0.364	-0.236		-0.360		
ASP	QM	-0.296	0.182				
	QM/MM	-0.396	0.206				
ASH	QM	-0.270	0.248				
	QM/MM	-0.358	0.265				
ASN	QM	-0.301	0.193				
	QM/MM	-0.362	0.163				
GLU	QM	-0.208	-0.359	0.159			
	QM/MM	-0.418	-0.320	0.186			
GLH	QM	-0.232	-0.263	0.229			
	QM/MM	-0.359	-0.251	0.251			
GLN	QM	-0.270	-0.311	0.204			
	QM/MM	-0.367	-0.259	0.152			
ARG	QM	-0.243	-0.290	-0.185		0.275	
	QM/MM	-0.318	-0.263	-0.174		0.278	
LYS	QM	-0.231	-0.283	-0.274	-0.185		
	QM/MM	-0.308	-0.249	-0.270	-0.185		
LYN	QM	-0.209	-0.299	-0.223	-0.144		
	QM/MM	-0.367	-0.255	-0.213	-0.141		
HIE	QM	-0.265	0.022	-0.221	-0.120		
	QM/MM	-0.365	0.067	-0.222	-0.115		
HID	QM	-0.240	-0.024	-0.249	-0.146		
	QM/MM	-0.339	-0.024	-0.237	-0.125		

Table S9: Mulliken partial charges q in units of the elementary charge e of side chain carbon atoms for each of the 22 amino acids obtained at the GFN2-xTB level. In case more than one carbon atom is present at a given position of a particular AA side chain, the partial charges are provided for the individual atoms.

		C_β	C_γ	C_δ	C_ϵ	C_ζ	C_η
ALA	QM<br>QM/MM	-0.115<br>-0.154					
VAL	QM<br>QM/MM	-0.019<br>-0.042	-0.107/-0.112<br>-0.103/-0.102				
LEU	QM<br>QM/MM	-0.072<br>-0.103	-0.012<br>-0.003	-0.109/-0.109<br>-0.106/-0.107			
ILE	QM<br>QM/MM	-0.025<br>-0.047	-0.106/-0.059<br>-0.102/-0.052	-0.104<br>-0.102			
PHE	QM<br>QM/MM	-0.076<br>-0.104	0.010<br>0.017	-0.038/-0.046<br>-0.039/-0.042	-0.028/-0.031<br>-0.03/-0.032	-0.033<br>-0.034	
TRP	QM<br>QM/MM	-0.074<br>-0.099	-0.036<br>-0.028	-0.01/-0.025<br>-0.012/-0.022	0.044/-0.04<br>0.043/-0.038	-0.053/-0.042<br>-0.054/-0.043	-0.036<br>-0.037
TYR	QM<br>QM/MM	-0.078<br>-0.104	-0.001<br>0.006	-0.04/-0.047<br>-0.04/-0.042	-0.07/-0.056<br>-0.072/-0.056	0.144<br>0.143	
SER	QM<br>QM/MM	0.075<br>0.042					
THR	QM<br>QM/MM	0.119<br>0.088	-0.122<br>-0.119				
CYS	QM<br>QM/MM	-0.007<br>-0.043					
MET	QM<br>QM/MM	-0.064<br>-0.101	-0.007<br>0.003		-0.052<br>-0.049		
ASP	QM<br>QM/MM	-0.101<br>-0.113	0.368<br>0.368				
ASH	QM<br>QM/MM	-0.101<br>-0.143	0.363<br>0.367				
ASN	QM<br>QM/MM	-0.102<br>-0.140	0.287<br>0.291				
GLU	QM<br>QM/MM	-0.049<br>-0.068	-0.104<br>-0.097	0.372<br>0.370			
GLH	QM<br>QM/MM	-0.062<br>-0.097	-0.095<br>-0.086	0.363<br>0.363			
GLN	QM<br>QM/MM	-0.063<br>-0.098	-0.093<br>-0.084	0.288<br>0.287			
ARG	QM<br>QM/MM	-0.076<br>-0.111	-0.075<br>-0.066	-0.017<br>-0.016		0.239<br>0.240	
LYS	QM<br>QM/MM	-0.075<br>-0.110	-0.070<br>-0.060	-0.082<br>-0.082	-0.034<br>-0.034		
LYN	QM<br>QM/MM	-0.068<br>-0.099	-0.062<br>-0.053	-0.066<br>-0.066	0.025<br>0.026		
HIE	QM<br>QM/MM	-0.077<br>-0.109	0.057<br>0.061	-0.034<br>-0.033	0.090<br>0.087		
HID	QM<br>QM/MM	-0.075<br>-0.106	0.024<br>0.028	-0.002<br>0.001	0.087<br>0.086		
